# Association between red blood cell distribution width and mortality in patients with metastatic brain tumors: A retrospective single-center cohort study

**DOI:** 10.3389/fonc.2022.985263

**Published:** 2022-10-07

**Authors:** Ji-Hoon Sim, Yong-Seok Park, Seungil Ha, Sung-Hoon Kim, Joung Uk Kim

**Affiliations:** Department of Anesthesiology and Pain Medicine, Asan Medical Center, University of Ulsan College of Medicine, Seoul, South Korea

**Keywords:** brain, metastases, survival, red blood cell distribution width, prognosis

## Abstract

Metastatic brain tumor has been associated with high mortality and poor prognosis. However, information on indicators predicting surgical prognosis in patients with brain metastases is limited. This study aimed to investigate the association between preoperative red blood cell distribution width (RDW) and mortality in patients who underwent surgery for metastatic brain tumors. This study analyzed 282 patients who underwent metastatic brain tumor surgery between August 1999 and March 2020. Patients were divided into two groups based on preoperative RDW cut-off values (<13.2 and ≥13.2). The surgical outcomes were compared between the two groups. Additionally, we performed Cox regression analysis to assess the association between preoperative RDW and 1-year and overall mortality. There were significant differences in 180-day mortality (6.2% vs. 28.7%, P<0.001), 1-year mortality (23.8% vs. 46.7%, P<0.001), and overall mortality (75.0% vs. 87.7%, P=0.012) between the two groups. In the Cox regression analysis, RDW ≥ 13.2 was significantly associated with higher 1-year mortality (adjusted hazard ratio [HR], 2.14; 95% confidence interval [CI], 1.38–3.30; P<0.001) and overall mortality (HR, 1.44; 95% CI, 1.09–1.90; P=0.010). Preoperative RDW is strongly associated with high mortality in metastatic brain tumor surgery.

## Introduction

Metastatic brain tumors are one of the major causes of high mortality and poor prognosis in patients with terminal cancer (1, 2). Brain metastases are also often accompanied by neurocognitive deterioration, such as decreased sensory and motor function, which is associated with poor quality of life (3). Treatment options for patients with brain metastases include surgical tumor resection, chemotherapy, stereotactic radiosurgery (SRS), whole-brain radiation therapy, and targeted therapy (4–6). The main goals of these treatments are to achieve local control of metastatic lesions, improve quality of life, and prevent death from neurological complications (7). However, despite these treatments, the median overall survival rate ranges from 2 to 27.3 months (3, 8–11), indicating an extremely poor prognosis. Although there have been some studies on the predictive prognosis in patients with brain metastasis (12, 13), information on this is still limited.

The red blood cell (RBC) distribution width (RDW) is a value of the variation in the size of RBCs in blood (14) and is closely related to acute and chronic inflammation (15, 16) as well as an indicator of anemia (17). Recently, RDW has been reported as a simple and objective indicator of patient survival and complications in acute and chronic diseases (18, 19). Several studies have also reported that preoperative RDW is associated with prognosis in various disease-related procedures (20, 21).

However, few comprehensive studies have been conducted on the associations between preoperative RDW and surgical prognosis in patients who underwent metastatic brain tumor surgery. Therefore, this study aimed to comprehensively evaluate the association between preoperative RDW and 1-year and overall mortality in patients who underwent surgery for metastatic brain tumors.

## Materials and methods

### Study design and population

This retrospective study was approved by the Asan Medical Center Institutional Review Committee (Protocol No. 2022-0596). Due to the retrospective nature of our study, the requirement for written informed consent was waived. We analyzed all the patients who underwent metastatic brain tumor surgery between August 1999 and March 2020. The following patients were excluded: patients aged <18 or ≥80 years; patients with hematologic disorders; patients who underwent emergency surgery; and patients with incomplete data or missing RDW values.

### Clinical data collection and study outcome

Patient demographic and perioperative variables were collected using the electronic medical record system. Demographic variables included age, height, weight, sex, body mass index (BMI), diabetes mellitus, hypertension, cardiovascular disease, chronic kidney disease, other diseases, the American Society of Anesthesiologists (ASA) classification, Karnofsky Performance Status (KPS) grading, postoperative chemotherapy, postoperative radiation therapy, and anticoagulant use. The KPS grading is a widely used standard method to assess the functional status of patients with cancer (22).

Variables related to patients’ cancer origin included breast, colorectal, liver, lung, skin, stomach, neck, unknown, and multiple organs. Tumor location and tumor maximum size variables were also collected.

Preoperative laboratory values included international normalized ratio and levels of hemoglobin, platelet, white blood cells, serum creatinine, albumin, neutrophil to lymphocyte ratio, C-reactive protein, carcinoembryonic antigen, carbohydrate antigen 19-9, sodium, potassium, chloride, aspartate transaminase, and alanine transaminase. The patient’s total blood count was determined as the closest laboratory test value to the date of surgery within 7 days before surgery in the ward.

Intraoperative variables included operative time, administered crystalloids, mannitol, urine output, and RBC transfusion.

The study outcomes were 180-day mortality rate (calculated from the date of surgery to 180-day follow-up), 1-year mortality rate (calculated from the date of surgery to 1-year follow-up), and overall mortality rate (determined from the date of surgery to the last follow-up) between the two groups divided according to preoperative RDW cut-off value. Cox regression analysis was also performed to assess the association between preoperative RDW and 1-year mortality and overall mortality. Additionally, preoperative RDW values between the survival and non-survival groups were compared at 180-day, 1-year, and overall period.

### Statistical analysis

Data are described as means ± SD, medians (interquartile ranges), or numbers (proportions), as appropriate. We used a Chi-square test or Fisher’s exact test for categorical data and Student’s t-test or Mann–Whitney U-test for continuous data. We performed a receiver operating characteristic (ROC) curve analysis to determine the cut-off level for predicting 1-year mortality. Cox regression analysis was performed to assess the association between preoperative RDW and mortality at 1 year and overall. All variables with P-value <0.1 in the univariate analysis were included in the multivariate analysis. The Kaplan–Meier method was used to evaluate 1-year and overall cumulative survival according to the preoperative RDW cut-off level. The log-rank test was used to evaluate changes between curves. A P-value <0.05 was regarded as statistically significant. All data were analyzed using SPSS Statistics for Windows (version 22.0; IBM Corp., Armonk, NY, USA) or R (version 3.1.2; R Foundation for Statistical Computing, Vienna, Austria).

## Results

**Figure 1:**
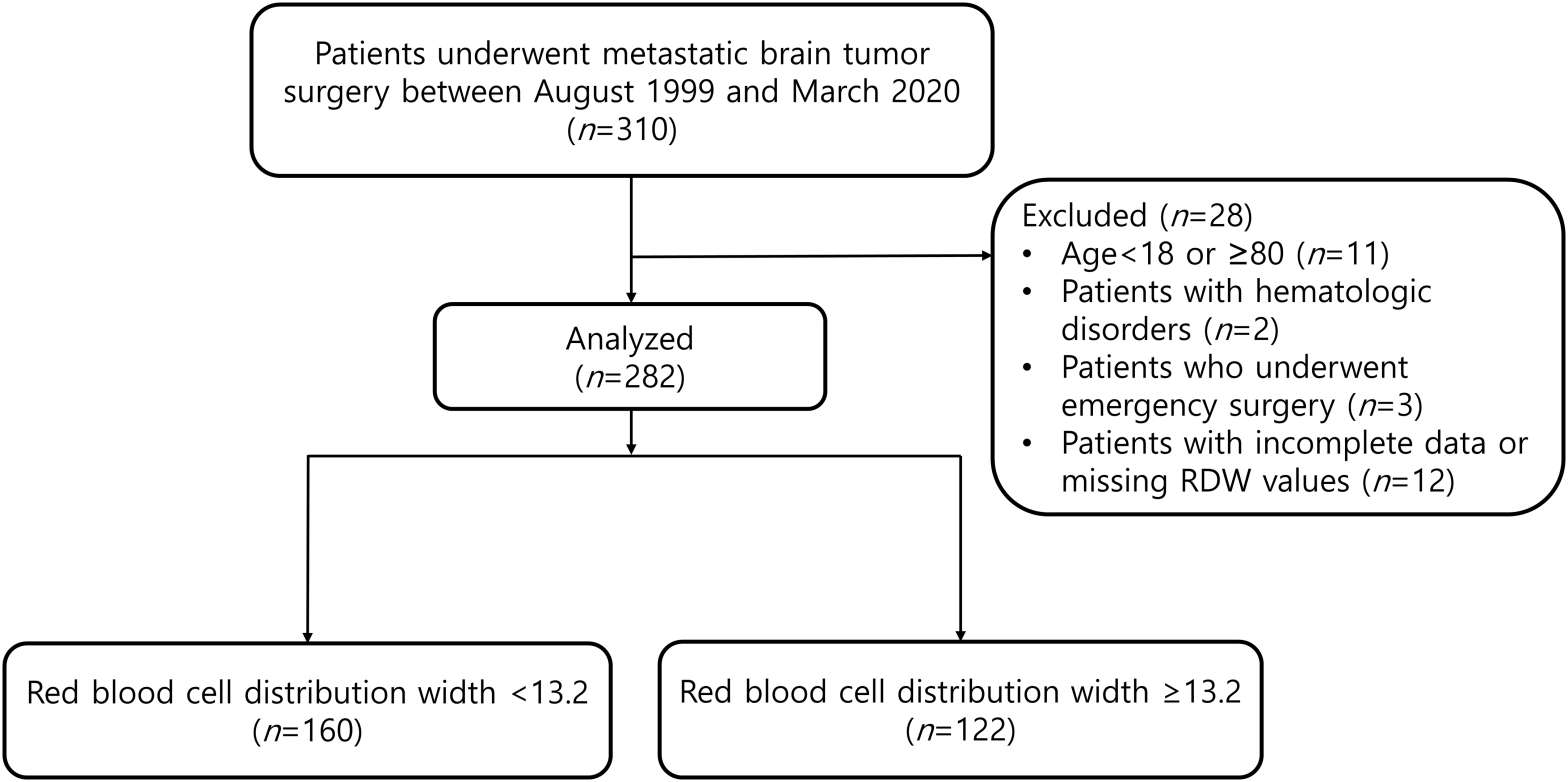
Study flowchart.

Of the 310 patients who underwent metastatic brain tumor surgery, 28 patients were excluded according to the exclusion criteria (**Figure 1**). The median follow-up time of patients for determining the overall mortality was 1.74 (0.75 to 3.88) years. According to the ROC curve analysis, a preoperative RDW cut-off value of 13.2 predicted 1-year mortality (area under the curve, 0.656; sensitivity, 60.0%; specificity, 65.2%). A total of 282 patients were divided into two groups: RDW <13.2 (n=160) and RDW ≥13.2 (n=122) (**Figure 1**).

The baseline characteristics and perioperative variables of the patients are shown in **Table 1**. There were no significant differences in demographic variables, such as age (P=0.514), sex (P=1.000), BMI (P=0.114), KPS grade (P=0.419), postoperative chemotherapy (P=0.183), postoperative radiation therapy (P=0.104), and ASA classification (P=0.052) between the two groups (**Table 1**). With respect to cancer-related variables, such as tumor location (P=0.713) and tumor maximum size (P=0.883), no significant differences were found between the two groups (**Table 1**).

**Table 1:** Baseline characteristics and perioperative variables of the study population.

	Study population			
	RDW<13.2	RDW≥13.2	Total	P
	(n = 160)	(n = 122)	(n = 282)	
**Demographic variables**				
Age, year	60.0 (52.0–68.5)	61.5 (54.0–68.0)	60.5 (52.0–68.0)	0.514
Height, cm	162.5 ± 7.6	162.4 ± 7.4	162.4 ± 7.5	0.902
Weight, kg	60.0 (54.4–67.9)	59.0 (52.8–67.0)	60.0 (53.9–67.1)	0.184
Sex, male	81 (50.6%)	61 (50.0%)	142 (50.4%)	1.000
BMI	23.2 (21.1–25.3)	22.5 (20.8–24.8)	22.9 (20.9–25.1)	0.114
DM	20 (12.5%)	16 (13.1%)	36 (12.8%)	1.000
HTN	38 (23.8%)	22 (18.0%)	60 (21.3%)	0.310
CVD	4 (2.5%)	4 (3.3%)	8 (2.8%)	0.977
CKD	0 (0.0%)	2 (1.6%)	2 (0.7%)	0.363
Others	7 (4.4%)	6 (4.9%)	13 (4.6%)	1.000
ASA				0.052
1	6 (3.8%)	5 (4.1%)	11 (3.9%)	
2	136 (85.0%)	89 (73.0%)	225 (79.8%)	
3	18 (11.2%)	27 (22.1%)	45 (16.0%)	
4	0 (0.0%)	1 (0.8%)	1 (0.4%)	
Karnofsky Performance Status				0.419
A (80–100)	134 (83.8%)	95 (77.9%)	229 (81.2%)	
B (40–80)	20 (12.5%)	22 (18.0%)	42 (14.9%)	
C (0–40)	6 (3.8)	5 (4.1%)	11 (3.9%)	
Postoperative chemotherapy	90 (43.8%)	58(47.5%)	148 (52.5%)	0.183
Postoperative radiation therapy	85 (53.1%)	52 (42.6%)	137 (48.6%)	0.104
Anticoagulant	1 (0.6%)	3 (2.5%)	4 (1.4%)	0.434
**Primary cancer origin**				
Breast	26 (16.2%)	20 (16.4%)	46 (16.3%)	1.000
Colorectal	8 (5.0%)	10 (8.2%)	18 (6.4%)	0.400
Kidney	9 (5.6%)	8 (6.6%)	17 (6.0%)	0.941
Liver	6 (3.8%)	9 (7.4%)	15 (5.3%)	0.282
Lung	51 (31.9%)	39 (32.0%)	90 (31.9%)	1.000
Skin	6 (3.8%)	1 (0.8%)	7 (2.5%)	0.238
Stomach	4 (2.5%)	5 (4.1%)	9 (3.2%)	0.678
Neck	2 (1.2%)	5 (4.1%)	7 (2.5%)	0.256
Unknown	21 (13.1%)	14 (11.5%)	35 (12.4%)	0.815
Multiple brain tumors	4 (2.5%)	6 (4.9%)	10 (3.5%)	0.446
Tumor location				0.713
Infratentorial	36 (22.5%)	29 (23.8%)	65 (23.0%)	
Supratentorial	122 (76.2%)	90 (73.8%)	212 (75.2%)	
Others	2 (1.2%)	3 (2.5%)	5 (1.8%)	
Tumor maximum size, cm	4.0 (3.1–5.0)	4.0 (3.0–5.4)	4.0 (3.0–5.2)	0.883
**Laboratory variables**				
Hemoglobin, g/dL	13.8 (12.8–14.7)	13.1 (11.8–13.8)	13.4 (12.4–14.4)	< 0.001
Platelet,10^9^/L	224.5 (191.0–283.0)	232.0 (183.0–302.0)	228.0 (190.0–289.0)	0.704
WBC,10^3^/uL	7.1 (5.5–9.4)	7.8 (6.2–11.3)	7.4 (5.8–10.1)	0.022
RBC, 10^6^/uL	4.5 ± 0.4	4.2 ± 0.5	4.3 ± 0.5	<0.001
PT, INR	1.0 (0.9–1.0)	1.0 (0.9–1.1)	1.0 (0.9–1.0)	0.565
Creatinine, mg/dL	0.8 (0.6–0.9)	0.8 (0.6–0.9)	0.8 (0.6–0.9)	0.239
Albumin, g/dL	3.9 (3.6–4.2)	3.6 (3.2–4.0)	3.8 (3.4–4.1)	< 0.001
NLR	3.8 (1.7–7.2)	4.5 (2.5–10.1)	4.0 (2.0–8.1)	0.082
CRP, mg/dL	0.1 (0.1–0.4)	0.1 (0.1–0.5)	0.1 (0.1–0.5)	0.278
CEA, ng/mL	1.9 (0.9–3.6)	1.9 (1.2–3.6)	1.9 (1.0–3.6)	0.742
CA 19-9, U/mL	8.5 (3.9–20.0)	14.4 (5.8–27.4)	10.1 (4.2–22.2)	0.065
Sodium	142.6 ± 3.6	141.6 ± 4.3	142.2 ± 4.0	0.048
Potassium	4.2 (4.0–4.5)	4.2 (4.0–4.5)	4.2 (4.0–4.5)	0.830
Chloride	111.0 (108.0–114.0)	109.5 (107.0–113.0)	110.0 (107.0–114.0)	0.070
AST, IU/L	22.0 (17.0–28.5)	22.0 (17.0–30.0)	22.0 (17.0–29.0)	0.491
ALT, IU/L	19.0 (14.0–26.5)	22.0 (14.0–37.0)	19.5 (14.0–31.0)	0.057
**Intraoperative variables**				
Operative time, min	300.0 (262.5–385.0)	300.0 (240.0–367.0)	300.0 (250.0–377.0)	0.404
Crystalloids, mL	2200.0 (1800.0–2800.0)	2150.0 (1700.0–2800.0)	2200.0 (1800.0–2800.0)	0.542
Mannitol, mL	100.0 (0.0–150.0)	100.0 (0.0–150.0)	100.0 (0.0–150.0)	0.615
Urine output, mL/kg/h	4.3 (2.9–5.8)	4.4 (2.7–5.8)	4.3 (2.8–5.8)	0.773
RBC transfusion	20 (12.5%)	25 (20.5%)	45 (16.0%)	0.099

RDW, red blood cell distribution width; BMI, body mass index; DM, diabetes mellitus; HTN, hypertension; CVD, cardiovascular disease; CKD, chronic kidney disease; ASA, American Society of Anesthesiologists; WBC, white blood cell; PT, prothrombin time; INR, international normalized ratio; NLR, neutrophil to lymphocyte ratio; CRP, C-reactive protein; CEA, carcinoembryonic antigen; CA 19-9, carbohydrate antigen 19-9; AST, aspartate aminotransferase; ALT, alanine aminotransferase; RBC, red blood cell

Values are expressed as means ± standard deviations, medians (interquartile ranges), or absolute numbers (percentages).

In terms of laboratory variables, the RDW ≥13.2 group had significantly lower hemoglobin (P<0.001), albumin (P<0.001), and sodium (P=0.048) levels and higher white blood cell count (P=0.022) (**Table 1**).

Moreover, no significant differences were found in the intraoperative variables, such as operative time (P=0.404), administered crystalloids (P=0.542), and RBC transfusion (P=0.099) between the two groups (**Table 1**).

### Study outcomes

Of the 282 patients, 45 (16.0%) expired within 180-day, 95 (33.7%) expired within 1 year, and 227 (80.5%) expired during the overall period (**Table 2**). The two groups showed significant differences in surgical outcomes, with the RDW ≥13.2 group demonstrating significantly higher rates of 180-day mortality (P<0.001), 1-year mortality (P<0.001), and overall mortality (P=0.012) (**Table 2**).

**Table 2:** Surgical outcomes of study population.

	Study population			
	RDW<13.2	RDW≥13.2	Total	P
	(n = 160)	(n = 122)	(n = 282)	
**Surgical outcomes**				
Hospital stay (days)	7.0 (5.0–10.0)	6.0 (5.0–8.0)	7.0 (5.0–10.0)	0.255
180-day mortality	10 (6.2%)	35 (28.7%)	45 (16.0%)	< 0.001
1-year mortality	38 (23.8%)	57 (46.7%)	95 (33.7%)	< 0.001
Overall mortality	120 (75.0%)	107 (87.7%)	227 (80.5%)	0.012

RDW, red blood cell distribution width.

Values are expressed as means ± standard deviations, medians (interquartile ranges), or absolute numbers (percentages).

In the Cox regression analysis, preoperative RDW ≥13.2 was significantly associated with 1-year mortality (hazard ratio [HR], 2.14; 95% confidence interval [CI], 1.38–3.30; P<0.001) (**Table 3**) and overall mortality (HR, 1.44; 95% CI, 1.09–1.90; P=0.010) (**Table 4**). Additionally, ASA classification 3 and 4 (HR, 2.05; 95% CI, 1.23–3.35; P=0.004), KPS grade (HR, 2.25; 95% CI, 1.39–3.64; P<0.001), postoperative chemotherapy (HR, 0.58; 95% CI, 0.38–0.88; P=0.010), liver cancer origin (HR, 4.14, 95% CI 1.92–8.92, P<0.001), and lung cancer origin (HR 2.49, 95% CI 1.59–3.88, P<0.001) were significantly associated with 1-year mortality (**Table 3**), whereas the overall mortality was significantly associated with male sex (HR, 1.44; 95% CI, 1.09–1.91, P=0.011), KPS grade (HR, 1.77; 95% CI, 1.26–2.49; P=0.001), liver cancer origin (HR, 2.52; 95% CI, 1.41–4.48; P=0.002), lung cancer origin (HR, 1.63; 95% CI, 1.21–2.20; P=0.001), and tumor maximum size (HR, 1.10; 95% CI, 1.01–1.20; P=0.036) (**Table 4**).

**Table 3:** Cox regression analyses of 1-year mortality.

	Univariate			Multivariable		
	HR	95% CI	*P*	HR	95% CI	*P*
RDW≥13.2	2.47	1.64–3.72	< 0.001	2.14	1.38–3.30	< 0.001
Age	1.01	0.99–1.03	0.290	0.99	0.97–1.01	0.221
BMI	0.98	0.92–1.04	0.434			
Sex (male)	1.78	1.18–2.69	0.006	1.55	0.98–2.43	0.059
ASA						
1, 2	1.00			1.00		
3, 4	2.21	1.39–3.51	< 0.001	2.05	1.23–3.35	0.004
DM	0.76	0.40–1.47	0.415			
HTN	0.78	0.46–1.31	0.346			
CVD	1.06	0.34–3.35	0.920			
CKD	1.49	0.21–10.69	0.692			
Karnofsky Performance Status						
A	1.00			1.00		
B, C	2.01	1.29–3.15	0.002	2.25	1.39–3.64	< 0.001
Postoperative chemotherapy	0.58	0.38–0.87	0.008	0.58	0.38–0.88	0.010
Postoperative radiation therapy	0.77	0.51–1.15	0.199			
**Primary cancer origin**						
Breast	0.82	0.47–1.45	0.492			
Colorectal	0.61	0.23–1.67	0.340			
Kidney	0.84	0.34–2.07	0.704			
Liver	2.92	1.47–5.82	0.002	4.14	1.92–8.92	< 0.001
Lung	2.10	1.41–3.15	< 0.001	2.49	1.59–3.88	< 0.001
Skin	0.75	0.19–3.06	0.694			
Stomach	1.48	0.54–4.02	0.447			
Neck	0.38	0.05–2.70	0.331			
Unknown	0.42	0.18–0.96	0.040			
Multiple brain tumors	1.76	0.71–4.33	0.221			
Tumor location						
Infratentorial	1.00					
Supratentorial	1.26	0.75–2.11	0.382			
Others	2.40	0.71–8.14	0.161			
Tumor maximum size, cm	1.11	0.98–1.27	0.111	1.10	0.96–1.26	0.167
Anemia (Hb <12 g/dL)	2.00	1.29–3.10	0.002	1.32	0.84–2.10	0.231
Hypoalbuminemia (albumin <3.5 g/dL)	1.81	1.19–2.76	0.005	1.01	0.64–1.59	0.971
NLR	1.02	1.00–1.04	0.104			
RBC transfusion	1.45	0.88–2.40	0.148			

HR, hazard ratio; CI, confidence interval; RDW, red blood cell distribution width; BMI, body mass index; DM, diabetes mellitus; HTN, hypertension; CVD, cardiovascular disease; CKD, chronic kidney disease; ASA, American Society of Anesthesiologists; NLR, neutrophil-to-lymphocyte ratio; RBC, red blood cell.

Values are expressed as means ± standard deviations, medians (interquartile ranges), or absolute numbers (percentages).

**Table 4:** Cox regression analyses of overall mortality.

	Univariate			Multivariable		
	HR	95% CI	*P*	HR	95% CI	*P*
RDW≥13.2	1.55	1.19–2.01	0.001	1.44	1.09–1.90	0.010
Age	1.01	0.99–1.02	0.424	0.99	0.98–1.00	0.146
BMI	1.00	0.97–1.04	0.857			
Sex (male)	1.54	1.19–2.00	0.001	1.44	1.09–1.91	0.011
ASA						
1, 2	1.00			1.00		
3, 4	1.51	1.07–2.12	0.019	1.42	0.99–2.03	0.059
DM	1.14	0.78–1.66	0.493			
HTN	1.15	0.84–1.56	0.379			
CVD	0.96	0.43–2.16	0.923			
CKD	0.58	0.08–4.16	0.590			
Karnofsky Performance Status						
A	1.00			1.00		
B, C	1.57	1.14–2.17	0.006	1.77	1.26–2.49	0.001
Postoperative chemotherapy	1.07	0.82–1.39	0.602	1.10	0.84–1.44	0.491
Postoperative radiation therapy	1.12	0.86–1.45	0.401			
**Primary cancer origin**						
Breast	0.99	0.69–1.42	0.962			
Colorectal	1.20	0.73–1.96	0.477			
Kidney	0.97	0.57–1.67	0.916			
Liver	2.13	1.24–3.67	0.006	2.52	1.41–4.48	0.002
Lung	1.59	1.21–2.09	<0.001	1.63	1.21–2.20	0.001
Skin	1.95	0.91–4.18	0.084			
Stomach	0.94	0.42–2.12	0.884			
Neck	0.66	0.27–1.60	0.355			
Unknown	0.56	0.37–0.86	0.008			
Multiple brain tumors	1.46	0.75–2.84	0.267			
Tumor location						
Infratentorial	1.00					
Supratentorial	1.07	0.79–1.46	0.655			
Others	0.68	0.21–2.17	0.510			
Tumor maximum size, cm	1.10	1.02–1.20	0.022	1.10	1.01–1.20	0.036
Anemia (Hb <12 g/dL)	1.65	1.21–2.24	0.001	1.27	0.92–1.77	0.147
Hypoalbuminemia (albumin <3.5 g/dL)	1.41	1.06–1.89	0.019	1.03	0.76–1.41	0.836
NLR	1.01	0.99–1.02	0.405			
RBC transfusion	1.12	0.79–1.59	0.510			

HR, hazards ratio; CI, confidence interval; RDW, red blood cell distribution width; BMI, body mass index; DM, diabetes mellitus; HTN, hypertension; CVD, cardiovascular disease; CKD, chronic kidney disease; ASA, American Society of Anesthesiologists; NLR, neutrophil-to-lymphocyte ratio; RBC, red blood cell.

Values are expressed as means ± standard deviations, medians (interquartile ranges), or absolute numbers (percentages).

**Figure 2:**
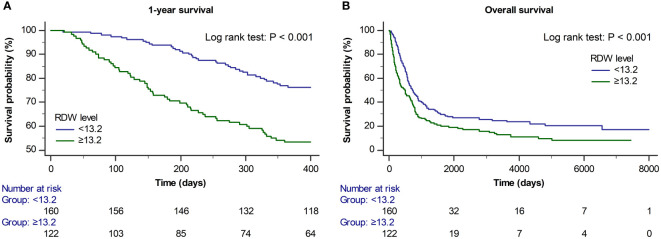
Kaplan–Meier curves for **(A)** 1-year and **(B)** overall survival according to the preoperative RDW cut-off value (<13.2 and ≥13.2) (log-rank test: *P* < 0.001 for 1-year survival and *P* < 0.001 for overall survival).

**Figure 2** shows the Kaplan–Meier curves of 1-year and overall mortality rates according to the preoperative RDW cut-off level (<13.2 and ≥13.2). The 1-year and overall mortality rates were significantly higher in the preoperative RDW ≥13.2 group than in the RDW <13.2 group (log-rank test: P<0.001 for 1-year mortality and P<0.001 for overall mortality).

Preoperative RDW values were significantly different between the survival and non-survival groups at 180-day (P<0.001), 1-year (P<0.001), and overall period (P=0.004) (**Supplementary Table 1**).

## Discussion

In this study, we found significant differences in 180-day, 1-year, and overall mortality rates according to the preoperative RDW cut-off value (<13.2 and ≥13.2) in patients who underwent surgery for metastatic brain tumor. Additionally, the preoperative RDW ≥13.2 was significantly associated with 1-year and overall mortality. These findings indicate that preoperative RDW might independently predict surgical prognosis in metastatic brain tumor surgery.

Despite recent advances in neurosurgical techniques, radiation therapy, and chemotherapy, metastatic brain tumors remain a major problem with a fatal impact on the prognosis of patients with cancer: relatively low median survival (2.9 months) (23) and 2-year survival rate (8%) (24). However, perioperative parameters that can predict mortality after metastatic brain tumor surgery are still limited. Tabouret et al. suggested that the number of systemic metastases and postoperative systemic treatment strategies is associated with better surgical outcome in brain metastases from breast cancer (25). Stankiewicz et al. reported that KPS grade, number of brain metastases, volume of largest lesion, and extra-cranial metastases were independent predictors of overall survival in robotic SRS (26). To the best of our knowledge, few studies have demonstrated the predictive power of other biomarkers in patients with metastatic brain tumor. This is the first study to comprehensively analyze the association between mortality rate and RDW in patients who underwent surgical resection for brain metastatic cancer and has clinical practicality considering that RDW is a relatively simple and inexpensive laboratory marker.

In our study, preoperative RDW ≥13.2 was significantly associated with 1-year and overall mortality. Although not fully understood, the following mechanisms may explain the strong association between RDW and postoperative mortality in metastatic brain tumor surgery.

First, increased RDW is a sign of a nutritional deficiencies and anemia, such as a deficiency of iron, folic acid, or vitamin B-12 (27). Malnutrition was associated with postoperative poor surgical prognosis in many previous studies (28, 29). An increase in preoperative RDW may also be associated with anemia and peripheral vascular disease (30), which may lead to intraoperative bleeding risk and blood transfusions (20, 31). Furthermore, transfusions may cause immunosuppression (32), hypothermia, and coagulopathy (33), which have been associated with mortality in surgical patients. Second, RDW is strongly associated with frailty (34, 35) and chronic inflammation (16, 36–38). Bone marrow suppression occurs during chronic inflammation, which leads to an increase in the RDW level due to an increase in the abnormal RBC production and anisocytosis (35). Therefore, RDW is one of several surrogate markers of chronic inflammation. Several studies have reported that chronic inflammation is associated with poor prognosis in various diseases and surgical patients (39, 40). Finally, oxidative stress that accompanies an increase in RDW may be a mechanism associated with postoperative mortality in patients (41). A recent study in elderly patients found that markers of serum oxidative stress were significantly associated with mortality (42).

In our study, KPS grade and liver and lung cancer origin were also significantly associated with 1-year and overall mortality in the Cox regression analysis. High KPS grade has been reported as a useful parameter for characterizing subgroups of patients with a more favorable prognosis (43). Generally, the life expectancy after a diagnosis of brain metastases from liver and lung cancers is extremely poor (44, 45).

This study has some limitations. First, our study has a retrospective design; therefore, the possibility of undocumented factors being reported, potential bias associated with patient selection, and recall bias exists. However, we attempted to reduce the influence of confounding factors by adjusting for variables that could affect the outcome. Second, there are no studies on the precise validation of preoperative RDW cut-off values that predict surgical prognosis in patients with brain metastases. Therefore, further well-designed studies of precise RDW cut-off values are needed. Additionally, several recent studies have reported that high RDW can be improved by nutritional supplements, diet, and exercise therapy (46–48). However, further large-scale studies are warranted to clarify whether nutritional supplementation and exercise therapy can improve RDW, as well as to clarify whether active interventions for RDW can improve outcomes in patients with high RDW. Lastly, our data were mostly collected from a single center comprising single ethnic groups in Korea, and the results may have been biased due to the homogeneous groups. Therefore, further multi-center studies with other ethnic groups are required.

In conclusion, we found that preoperative RDW ≥13.2 was strongly associated with 1-year and overall mortality in patients undergoing surgical resection for metastatic brain tumor. These results indicate that preoperative RDW can be a useful predictor of survival in patients with metastatic brain tumor.

## Data availability statement

The raw data supporting the conclusions of this article will be made available by the authors, without undue reservation.

## Ethics statement

The studies involving human participants were reviewed and approved by Asan Medical Center Institutional Review Committee. Written informed consent for participation was not required for this study in accordance with the national legislation and the institutional requirements.

## Author contribution

All authors contributed to the study conception and design. J-HS, Y-SP, and S-HK conceived and designed the study; J-HS and Y-SP were involved in data acquisition; J-HS, SH, and JK were involved in analysis and/or interpretation of data; J-HS drafted the manuscript; S-HK revised the manuscript critically for important intellectual content. All authors contributed to the article and approved the submitted version.

## Funding

This research was supported by a grant of the Korea Health Technology R&D Project through the Korea Health Industry Development Institute (KHIDI), which is funded by the Ministry of Health & Welfare, Republic of Korea (grant number: HI18C2383), and was supported by Institute of Information & communications Technology Planning & Evaluation (IITP) grant funded by the Korea government (MSIT, Project No. 2021-0-00393). This study was also supported by a grant (2022IP0053) from the Asan Institute for Life Sciences, Asan Medical Center, Seoul, Korea.

## Conflict of interest

The authors declare that the research was conducted in the absence of any commercial or financial relationships that could be construed as a potential conflict of interest.

## Publisher’s note

All claims expressed in this article are solely those of the authors and do not necessarily represent those of their affiliated organizations, or those of the publisher, the editors and the reviewers. Any product that may be evaluated in this article, or claim that may be made by its manufacturer, is not guaranteed or endorsed by the publisher.

## References

[1] Hatiboglu MA Wildrick DM Sawaya R. The role of surgical resection in patients with brain metastases. Ecancermedicalscience. (2013) 7:308. doi: 10.3332/ecancer.2013.308 23634178 PMC3628720

[2] Liu Q Tong X Wang J. Management of brain metastases: history and the present. Chin neurosurgical J (2019) 5:1. doi: 10.1186/s41016-018-0149-0 PMC7398203 32922901

[3] Lee SS Ahn JH Kim MK Sym SJ Gong G Ahn SD. Brain metastases in breast cancer: prognostic factors and management. Breast Cancer Res Treat (2008) 111(3):523–30. doi: 10.1007/s10549-007-9806-2 17990100

[4] Gil-Gil MJ Martinez-Garcia M Sierra A Conesa G Del Barco S González-Jimenez S. Breast cancer brain metastases: a review of the literature and a current multidisciplinary management guideline. Clin Transl Oncol (2014) 16(5):436–46. doi: 10.1007/s12094-013-1110-5 PMC3983876 24277572

[5] Witzel I Oliveira-Ferrer L Pantel K Müller V Wikman H. Breast cancer brain metastases: biology and new clinical perspectives. Breast Cancer Res (2016) 18(1):8. doi: 10.1186/s13058-015-0665-1 26781299 PMC4717619

[6] Leone JP Leone BA. Breast cancer brain metastases: the last frontier. Exp Hematol Oncol (2015) 4:33. doi: 10.1186/s40164-015-0028-8 26605131 PMC4657380

[7] Hatiboglu MA Akdur K Sawaya R. Neurosurgical management of patients with brain metastasis. Neurosurgical review. (2020) 43(2):483–95. doi: 10.1007/s10143-018-1013-6 30058049

[8] Ogawa K Yoshii Y Nishimaki T Tamaki N Miyaguni T Tsuchida Y. Treatment and prognosis of brain metastases from breast cancer. J Neurooncol. (2008) 86(2):231–8. doi: 10.1007/s11060-007-9469-1 17849084

[9] Kavouridis VK Harary M Hulsbergen AFC Lo YT Reardon DA Aizer AA. Survival and prognostic factors in surgically treated brain metastases. J Neurooncol. (2019) 143(2):359–67. doi: 10.1007/s11060-019-03171-6 30989623

[10] Leone JP Lee AV Brufsky AM. Prognostic factors and survival of patients with brain metastasis from breast cancer who underwent craniotomy. Cancer Med (2015) 4(7):989–94. doi: 10.1002/cam4.439 PMC4529337 25756607

[11] Rastogi K Bhaskar S Gupta S Jain S Singh D Kumar P. Palliation of brain metastases: Analysis of prognostic factors affecting overall survival. Indian J palliative Care (2018) 24(3):308–12. doi: 10.4103/IJPC.IJPC_1_18 PMC6069611 30111944

[12] Huang Z Sun B Wu S Meng X Cong Y Shen G. A nomogram for predicting survival in patients with breast cancer brain metastasis. Oncol Lett (2018) 15(5):7090–6. doi: 10.3892/ol.2018.8259 PMC5920309 29725432

[13] Rostami R Mittal S Rostami P Tavassoli F Jabbari B. Brain metastasis in breast cancer: a comprehensive literature review. J Neurooncol. (2016) 127(3):407–14. doi: 10.1007/s11060-016-2075-3 26909695

[14] Salvagno GL Sanchis-Gomar F Picanza A Lippi G. Red blood cell distribution width: A simple parameter with multiple clinical applications. Crit Rev Clin Lab Sci (2015) 52(2):86–105. doi: 10.3109/10408363.2014.992064 25535770

[15] Bellan M Soddu D Zecca E Croce A Bonometti R Pedrazzoli R. Association between red cell distribution width and response to methotrexate in rheumatoid arthritis. Reumatismo. (2020) 72(1):16–20. doi: 10.4081/reumatismo.2020.1243 32292017

[16] Agarwal S. Red cell distribution width, inflammatory markers and cardiorespiratory fitness: results from the national health and nutrition examination survey. Indian Heart J (2012) 64(4):380–7. doi: 10.1016/j.ihj.2012.06.006 PMC3860736 22929821

[17] Uchida T. Change in red blood cell distribution width with iron deficiency. Clin Lab haematol (1989) 11(2):117–21. doi: 10.1111/j.1365-2257.1989.tb00193.x 2766669

[18] Parizadeh SM Jafarzadeh-Esfehani R Bahreyni A Ghandehari M Shafiee M Rahmani F. The diagnostic and prognostic value of red cell distribution width in cardiovascular disease; current status and prospective. Biofactors. (2019) 45(4):507–16. doi: 10.1002/biof.1518 31145514

[19] Wang PF Song SY Guo H Wang TJ Liu N Yan CX. Prognostic role of pretreatment red blood cell distribution width in patients with cancer: A meta-analysis of 49 studies. J Cancer (2019) 10(18):4305–17. doi: 10.7150/jca.31598 PMC6691718 31413750

[20] Lee KR Park SO Kim SY Hong DY Kim JW Baek KJ. Red cell distribution width as a novel marker for predicting high-risk from upper gastro-intestinal bleeding patients. PLoS One (2017) 12(11):e0187158. doi: 10.1371/journal.pone.0187158 29095860 PMC5667835

[21] Fatemi O Torguson R Chen F Ahmad S Badr S Satler LF. Red cell distribution width as a bleeding predictor after percutaneous coronary intervention. Am Heart J (2013) 166(1):104–9. doi: 10.1016/j.ahj.2013.04.006 23816028

[22] Péus D Newcomb N Hofer S. Appraisal of the karnofsky performance status and proposal of a simple algorithmic system for its evaluation. BMC Med Inf decision making (2013) 13:72. doi: 10.1186/1472-6947-13-72 PMC3722041 23870327

[23] Lamba N Kearney RB Catalano PJ Hassett MJ Wen PY Haas-Kogan DA. Population-based estimates of survival among elderly patients with brain metastases. Neuro-oncology. (2021) 23(4):661–76. doi: 10.1093/neuonc/noaa233 PMC8041328 33068418

[24] Chamberlain MC Baik CS Gadi VK Bhatia S Chow LQ. Systemic therapy of brain metastases: non-small cell lung cancer, breast cancer, and melanoma. Neuro-oncology (2017) 19(1):i1–i24. doi: 10.1093/neuonc/now197 28031389 PMC5193029

[25] Tabouret E Metellus P Tallet-Richard A Figarella-Branger D Charaffe-Jauffret E Viens P. Surgical resection of brain metastases from breast cancer in the modern era: clinical outcome and prognostic factors. Anticancer Res (2013) 33(5):2159–67. 23645770

[26] Stankiewicz M Tomasik B Blamek S. A new prognostic score for predicting survival in patients treated with robotic stereotactic radiotherapy for brain metastases. Sci Rep (2021) 11(1):20347. doi: 10.1038/s41598-021-98847-3 34645854 PMC8514560

[27] Sultana GS Haque SA Sultana T Ahmed AN. Value of red cell distribution width (RDW) and RBC indices in the detection of iron deficiency anemia. Mymensingh Med J (2013) 22(2):370–6. 23715364

[28] Correia MI Waitzberg DL. The impact of malnutrition on morbidity, mortality, length of hospital stay and costs evaluated through a multivariate model analysis. Clin Nutr (Edinburgh Scotland) (2003) 22(3):235–9. doi: 10.1016/S0261-5614(02)00215-7 12765661

[29] Soloff MA Vargas MV Wei C Ohnona A Tyan P Gu A. Malnutrition is associated with poor postoperative outcomes following laparoscopic hysterectomy. JSLS: J Soc Laparoendoscopic Surgeons (2021) 25(1):84–7. doi: 10.4293/JSLS.2020.00084 PMC8035827 33879999

[30] Satılmış S Karabulut A. Correlation between red cell distribution width and peripheral vascular disease severity and complexity. Med Sci (Basel Switzerland) (2019) 7(7):77. doi: 10.3390/medsci7070077 PMC6681184 31324033

[31] Sim JH Kwon HM Jun IG Kim SH Kim B Kim S. Association between red blood cell distribution width and blood transfusion in patients undergoing living donor liver transplantation: Propensity score analysis. J hepato-biliary-pancreatic Sci (2022). doi: 10.1002/jhbp.1163 35466566

[32] Babaev A Pozzi F Hare G Zhang H. Storage of red blood cells and transfusion-related acute lung injury. J Anesth Crit care: Open Access (2014) 1(1):00002. doi: 10.15406/jaccoa.2014.01.00002 PMC5218835 28066804

[33] Reynolds BR Forsythe RM Harbrecht BG Cuschieri J Minei JP Maier RV. Hypothermia in massive transfusion: have we been paying enough attention to it? J Trauma acute Care Surg (2012) 73(2):486–91. 23019675

[34] Li CM Chao CT Chen SI Han DS Huang KC. Elevated red cell distribution width is independently associated with a higher frailty risk among 2,932 community-dwelling older adults. Front Med (2020) 7:470. doi: 10.3389/fmed.2020.00470 PMC7477345 32984367

[35] Kim J Im JS Choi CH Park CH Lee JI Son KH. The association between red blood cell distribution width and sarcopenia in U. S. Adults Sci Rep (2018) 8(1):11484. doi: 10.1038/s41598-018-29855-z 30065297 PMC6068096

[36] Lappegård J Ellingsen TS Hindberg K Mathiesen EB Njølstad I Wilsgaard T. Impact of chronic inflammation, assessed by hs-CRP, on the association between red cell distribution width and arterial cardiovascular disease: The tromsø study. TH open: companion J to Thromb haemostasis (2018) 2(2):e182–e9. doi: 10.1055/s-0038-1651523 PMC6524874 31249941

[37] Schepens T De Dooy JJ Verbrugghe W Jorens PG. Red cell distribution width (RDW) as a biomarker for respiratory failure in a pediatric ICU. J Inflammation (London England) (2017) 14:12. doi: 10.1186/s12950-017-0160-9 PMC5463327 28596707

[38] Olafsson HB Sigurdarson GA Christopher KB Karason S Sigurdsson GH Sigurdsson MI. A retrospective cohort study on the association between elevated preoperative red cell distribution width and all-cause mortality after noncardiac surgery. Br J anaesthesia (2020) 124(6):718–25. doi: 10.1016/j.bja.2020.02.009 PMC7931157 32216958

[39] Abdullah HR Sim YE Sim YT Ang AL Chan YH Richards T. Preoperative red cell distribution width and 30-day mortality in older patients undergoing non-cardiac surgery: a retrospective cohort observational study. Sci Rep (2018) 8(1):6226. doi: 10.1038/s41598-018-24556-z 29670189 PMC5906451

[40] Lee SI Lee SY Choi CH Park CH Park KY Son KH. Relation between changes in red blood cell distribution width after coronary artery bypass grafting and early postoperative morbidity. J Thorac disease (2018) 10(7):4244–54. doi: 10.21037/jtd.2018.06.108 PMC6106013 30174870

[41] Tajuddin SM Nalls MA Zonderman AB Evans MK. Association of red cell distribution width with all-cause and cardiovascular-specific mortality in African American and white adults: a prospective cohort study. J Trans Med (2017) 15(1):208. doi: 10.1186/s12967-017-1313-6 PMC5640961 29029617

[42] Schöttker B Saum KU Jansen EH Boffetta P Trichopoulou A Holleczek B. Oxidative stress markers and all-cause mortality at older age: a population-based cohort study. journals gerontology Ser A Biol Sci Med Sci (2015) 70(4):518–24. doi: 10.1093/gerona/glu111 25070660

[43] Reali A Allis S Girardi A Verna R Bianco L Redda MG. Is karnofsky performance status correlate with better overall survival in palliative conformal whole brain radiotherapy? our experience. Indian J palliative Care (2015) 21(3):311–6. doi: 10.4103/0973-1075.164891 PMC4617039 26600700

[44] Ali A Goffin JR Arnold A Ellis PM. Survival of patients with non-small-cell lung cancer after a diagnosis of brain metastases. Curr Oncol (Toronto Ont) (2013) 20(4):e300–6. doi: 10.3747/co.20.1481 PMC3728058 23904768

[45] Han MS Moon KS Lee KH Cho SB Lim SH Jang WY. Brain metastasis from hepatocellular carcinoma: the role of surgery as a prognostic factor. BMC cancer (2013) 13:567. doi: 10.1186/1471-2407-13-567 24289477 PMC3879022

[46] Horne BD Muhlestein JB May HT Le VT Bair TL Bennett ST. Preferential metabolic improvement by intermittent fasting in people with elevated baseline red cell distribution width: A secondary analysis of the WONDERFUL randomized controlled trial. Nutrients (2021) 13(12):4407. doi: 10.3390/nu13124407 34959959 PMC8703681

[47] Loprinzi PD Hall ME. Physical activity and dietary behavior with red blood cell distribution width. Physiol behavior (2015) 149:35–8. doi: 10.1016/j.physbeh.2015.05.018 26003494

[48] Ryan JJ Hanes DA Bradley RD Contractor N. Effect of a nutrition support formula in adults with inflammatory bowel disease: A pilot study. Global Adv Health Med (2019) 8:2164956119867251. doi: 10.1177/2164956119867251 PMC6664624 31384513

