# Supplementary material for: Association between red blood cell distribution width and mortality in patients with metastatic brain tumors: A retrospective single-center cohort study
Source: Front Oncol. 2022 Oct 7;12:985263. doi: 10.3389/fonc.2022.985263 (PMC9586452; doi:10.3389/fonc.2022.985263)
Supplement: Supplementary file 1 [file Table_1.docx]

**Supplementary Table 1. Preoperative RDW between survival and non-survival groups at 180-days, 1-year, and overall period**

|  | **Survival group (n=237)** | **Non-survival group (n=45)** | ***P*** |
| --- | --- | --- | --- |
| Preoperative RDW | 12.9 (12.4–13.7)* | 14.0 (13.3–15.4)* | <0.001 |
|  | 12.9 (12.4–13.6)† | 13.5 (12.9–14.7)† | <0.001 |
|  | 12.8 (12.2–13.5)** | 13.2 (12.5–14.2)** | 0.004 |

*at 180 days, †at 1-year, **at overall period

RDW, red blood cell distribution width.

Values are expressed as medians (interquartile ranges).
